# Effect of Combined Electromagnetic Field and Plantar Flexion Resistance Exercise on Wound Healing in Patients with Venous Leg Ulcers: A Randomized Controlled Trial

**DOI:** 10.3390/medicina59061157

**Published:** 2023-06-15

**Authors:** Heba Mohamed Mohamady, Mona Mohamed Taha, Yasser M. Aneis, Monira I. Aldhahi, Asmaa Fawzy Attalla

**Affiliations:** 1 Department of Physical Therapy for Surgery, Faculty of Physical Therapy, Cairo University, Giza 11432, Egypt; 2 Department of Rehabilitation Sciences, College of Health and Rehabilitation Sciences, Princess Nourah bint Abdulrahman University, P.O. Box 84428, Riyadh 11671, Saudi Arabia; mialdhahi@pnu.edu.sa; 3 Department of Basic Sciences, Faculty of Physical Therapy, Cairo University, Giza 11432, Egypt; dryassercom@cu.edu.eg; 4 Department of Basic Sciences, Faculty of Physical Therapy, Delta University for Science and Technology, Gamasa City 11152, Egypt

**Keywords:** electromagnetic fields, resistance training, venous ulcer, wound healing

## Abstract

*Background and Objectives*: Venous ulcers are recognized to be more painful and resistant to therapy than ulcers of other etiologies. Various methods have been used for the conservative treatment of venous ulcers, such as pulsed electromagnetic field (PEMF) and plantar exercise, which promote wound healing due to a range of physiological effects. The study aimed to examine the effect of combined pulsed electromagnetic field therapy and plantar flexion resistance exercise (PRE) on patients with venous leg ulcers (VLUs). *Materials and Methods*: The study was a prospective, randomized controlled trial. A total of 60 patients between the ages of 40 and 55 with venous ulcers were randomly assigned to 1 of 3 groups. For up to 12 weeks, the first group received PEMF therapy and plantar flexion resistance exercise (PRE) therapy in addition to conservative ulcer treatment for up to 12 weeks. The second group received only PEMF therapy in addition to conservative ulcer treatment, while the third group served as the control and received only conservative ulcer treatment. *Results*: At the four-week follow-up, the two experimental groups revealed a considerable variation in ulcer surface area (USA) and ulcer volume (UV), with no significant change in the control group. At the 12-week follow-up, there were significant differences between the three groups, while group A underwent the most significant changes, with mean differences at [95% confidence interval] of (−4.75, −3.82, −0.98) for USA and (−12.63, −9.55, −2.45) for UV, respectively. *Conclusions*: On a short-term basis, adding a plantar resistance exercise to the PEMF had no appreciable short-term effects on ulcer healing; however, their combination had more pronounced medium-term effects.

## 1. Introduction

Venous or stasis ulcers account for 80% of leg ulcers [1]. They affect 1% of the population and contribute significantly to chronic wounds. Venous ulcers can be painful and have an impact on quality of life. They mostly develop along the medial distal part of the leg [2], and can remain open for weeks or years, while are frequently recurrent. Women and older people tend to develop venous ulcers more frequently [3]. Despite the low overall incidence, the refractory nature of those ulcers raises the risk of morbidity and mortality and has a significant negative impact on the quality of life of the patient [4].

Venous leg ulcers (VLUs) are caused by chronic venous insufficiency (CVI), or venous disease of the leg. CVI is a venous system malfunction caused by the failure of the calf muscle pump [5]. The calf muscle pump is composed of the calf muscle, superficial and deep veins, as well as a perforator vein. The outflow vein for this pump is the popliteal vein. When the calf muscle pump cannot lower the ambulatory venous pressure, a persistent increase in pressure after exercise occurs, known as ambulatory venous hypertension [6]. Ambulatory venous hypertension is believed to be the major pathologic cause of venous ulcers [7]. 

VLU is currently treated with local wound management, compression therapy, and other advanced treatments, such as bioengineered cell technology and negative pressure therapy. The gold standard for treating venous leg ulcers is compression therapy [8]. Compression therapy is essential for the treatment of venous leg ulcers as it improves venous return [9]. Compression has the physiologic benefits of increasing venous flow, decreasing venous reflux or edema, and increasing oxygenation in adjacent dermal skin tissue, as well as inducing fibrinolysis [7].

In fact, up to 15 to 30 percent of chronic VLUs do not improve with compression therapy [10] and continue to be non-healing after a year of therapy [11]. It implies that alternative adjuvant therapy needs to be investigated further to mitigate the impaired healing process.

Successful biofilm control is crucial for optimal wound management and healing.

Biofilms are communities of microorganisms that can form on wounds, impeding the immune system and antimicrobial treatments. They often complicate chronic wounds and are resistant to various treatments, making them challenging to manage [12]. When the biofilm is suspected, an appropriate dressing should be used to remove detached sections and absorb circulating cells. Biofilm eradication requires physical and chemical debridement, as well as the application of topical and systemic antimicrobials and dressings [13]. 

Wound care for ulcers includes several components, such as cleaning the wound, debridement, maintaining a moist environment, and using appropriate dressings [14]. The initial step in treating ulcers is to clean the area, while swabbing or irrigating the site are two typical cleaning procedures. Swabbing involves utilizing wet gauze to remove contaminants and dead tissue, whereas irrigation involves spraying the wound with a normal saline solution using a needle, syringe, or spraying device [15]. Maintaining a moist environment around the wound is important for promoting healing [16]. Reducing certain microbial species in chronic wounds can help reduce unpleasant odors caused by anaerobic bacteria or mixed communities of bacteria that impede healing. Several topical antimicrobial agents that are frequently used in wound care include fusidic acid, iodine, mupirocin, silver-containing products, and chlorhexidine [17]. Debridement, particularly mechanical debridement, may be beneficial in promoting the healing of chronic wounds by removing necrotic tissue [16].

Exercise has the potential to serve as both a therapeutic and preventive intervention. Additionally, there is evidence that physical inactivity can hinder healing [18]. It has been clearly shown that a calf training program can improve muscle endurance and even restore proper muscle pump function by increasing ejection fraction and decreasing residual fraction [19].

Numerous clinical studies have shown that electrical stimulation of the skin promotes wound healing by enhancing the endogenous currents induced by injury [20]. Pulsed electromagnetic fields (PEMFs) are an additional intervention that has been used in recent years, mainly in connection with fractures, burns, wound healing, and the treatment of various acute soft tissue injuries [21]. It is becoming more prevalent as an alternative treatment. Magnetic therapy can cut healing time by more than half [22]. In such conditions, magnet therapy not only helps with recovery but can also heal these conditions better, faster, and with less scar tissue. 

Venous ulcers can quickly get worse, placing the patient at risk for problems that can result in some people losing their limbs. However, it may be possible to prevent these problems with successful treatment. The authors hypothesized that combining plantar resistance exercise with PEMF could improve and accelerate the healing of venous leg ulcers. Limited studies have investigated the effect of the combined electromagnetic field and plantar resistance exercise therapy on individuals with venous leg ulcers. Therefore, the overarching aim of this study was to examine the effect of combining an electromagnetic field with plantar resistance exercise therapy on ulcer surface area (USA) and volume among patients with venous leg ulcers. The findings of this study can help patients with venous leg ulcers and healthcare providers by providing data on a non-invasive, effective treatment for venous leg ulcers.

## 2. Materials and Methods

### 2.1. Participants

The study was a three-arm, randomized controlled trial that was carried out on sixty patients (male and female) who had grade two primary venous ulcers, according to the venous clinical severity score. Patients were eligible for inclusion in the study if they had primary venous ulcers in the presence of venous insufficiency, as diagnosed by the site’s lead clinician and confirmed by clinical assessment and/or duplex ultrasound, in accordance with clinical practice guidelines by the Society for Vascular Surgery and the American Venous Forum [23]. Their ages ranged from 40 to 55 years. The study was conducted at the surgical department of El Mansoura Health Insurance Hospital, El Mansoura, Egypt. Ethical approval (REC/012/003629) was obtained by the Institutional Review Board of the Faculty of Physical Therapy, Cairo University. Patient rights and procedures were explained to the patient, and rewritten informed consent was completed prior to group allocation. This research was registered at ClinicalTrials.gov (NCT05410613). 

The patients included in this study were diagnosed with a grade 2 primary venous ulcer, according to the venous clinical severity score, which is made up of 10 features that are graded on a 4-point scale (absence (0), mild (1), moderate (2), and severe (3)). It has been demonstrated to be beneficial in assessing treatment responses in chronic venous diseases and is recommended for routine clinical usage in the clinical practice guideline [23,24,25]. The ankle/brachial index for all patients had to be greater than 0.80, implying appropriate arterial perfusion. The participants were randomized into three groups: In the PEMF+PRE group, the intervention program included using PEMF therapy in addition to PRE using a Step It rocker pedal (Step It System AB, Saltsjöbaden, Sweden) for up to 12 weeks; in the PEMF group, the intervention program included the use of PEMF therapy only, whereas the control group received conservative treatment for the ulcer only. 

Following the recruitment of the eligible patients, we randomly assigned the participants using a block randomization approach, according to a preset ratio of 1:1:1 block randomization approach. The participants were divided into three assigned groups (PEMF + PRE, PEMF, or control) in sequence. Participants, outcome evaluators, and data analysts were blind to the research participants’ group assignment. 

The following participants were excluded: peripheral vascular disease (PVD), arterial disease by ankle brachial pressure index (ABPI < 0.8), VLUs with infection, or cellulitis symptoms, as well as VLUs with necrotic tissue or slough or having more than one ulcer. Other ulcer types, included malignant ulceration, diabetic foot, and rheumatoid vasculitis; corticosteroid use; patients with dementia or who were disoriented; patients who had undertaken another physical therapy modality for ulcer healing. 

### 2.2. Outcome Measures

The outcome measures were assessed at three points during the timeline: at baseline, a four-week exercise training period, and a twelve-week period. The primary outcome measure was ulcer surface area. The secondary outcome measure was ulcer volume (UV) measurement in cm^3^ (width × length × depth). 

Primary outcome measures: The ulcer surface was measured by covering the ulcer with a piece of sterilized transparency film and using a fine-tipped transparency marker to trace the ulcer perimeter on the film. For each ulcer, a different transparency was employed. After that, the tracing was applied to metric graph paper, and the number of 1 mm that it contains was counted (only squares that were exactly 1 mm in size were counted inside the perimeter, and the area was transformed to square centimeters) [26].

Secondary outcome measures: ulcer volume measurement in cm^3^ (width × length × depth).

The ulcer was drawn on clear paper and placed over metric graph paper so that it would have the greatest length and width. Then, a disposable measuring tape was inserted into the ulcer’s bottom part to record the depth of the ulcer [27].

### 2.3. Interventions

In this study, the patients were randomly assigned into 1 of the 3 groups for 12 weeks of intervention (a sample of 20 patients in each group). They were randomly allocated to each group with an allocation ratio of 1:1:1 by blindly selecting numbers from sealed envelopes, created by a random block randomization technique. All subjects in the three groups received the same conservative treatment for the ulcer in the form of wound cleaning, dressing, debridement if needed by the physician, and compression therapy. The wound dressing was changed 2–3 times a week, following the local protocol. The ulcer was cleaned with regular saline solution, and a nonadherent dressing, such as Vaseline gauze, was used to maintain a moist environment. All participants in the study received the same medical treatment protocol, which included the administration of phlebotropic drugs in the form of flavonoid medications, as well as topical application of antibiotic or antimicrobial agents (octenidine dihydrochloride antiseptic) [28] and/or herbal products, in addition to analgesics, as needed [25]. Elastic compression stockings (Truform 0845; medical support hose; unisex style durable knit; 30–40 strength graduated compression) were used for compression therapy, which was removed at night, while patients were encouraged to elevate the affected limb.

PEMF + PRE group: Patients received a local application of pulsed electromagnetic field therapy (PEMF) and a plantar flexion resistance exercise (PRE) therapy by the Step It rocker pedal (Step It System AB, Saltsjöbaden, Sweden) for up to 12 weeks. The entire exercise session duration was 20 min and involved a 10-min interval period for pedaling with the index leg for 1 min, interspersed by passive recovery for 1 min. This routine was performed twice daily (e.g., morning and evening). There would be at least 300 pedals using the minimum recommended 2 s down and 2 s up space [29]. PEMF was provided using commercially available apparatus, the Magnetic Biostimulation Device MBS-system: (G-pulse 210 μp) applied via a coil. PEMF therapy was applied with an intensity equal to 3 mt and a frequency of magnetic field impulses equal to 4 Hz. All patients in the active treatment group received a 30 min treatment session, 3 days per week for 12 weeks, for a total of 36 sessions [30]. PEMF group: Patients received a local application of PEMF only, which was provided using commercially available apparatus, Magnetic Biostimulation Device MBS-system: (G-pulse 210 μp) applied by coil. Control group: patients received conservative treatment for the ulcer only. 

### 2.4. Sample Size and Statistical Analysis

**Figure 1:**
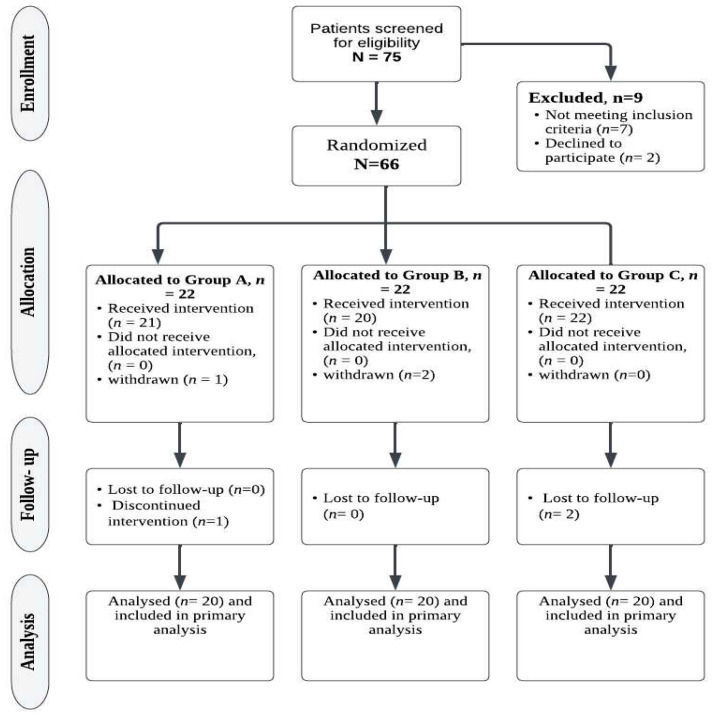
Consort diagram for the study.

The G*Power software was used to calculate the sample size (version 3.0.10, Germany). According to F tests (multivariate analysis of variance: MANOVA repeated measures, within–between interactions), a sample size of 60 patients was sufficient, with a Type I error of 0.05, a power of 80%, and an effect size of 0.37. The sample size for each group was increased by 5 patients to account for the dropout rate. The study’s consort diagram is described in Figure 1.

Before conducting the data analysis, the normality and homogeneity of variance were examined, and no deviations were found for any of the dependent variables, according to the results of the Shapiro–Wilk test and Levene’s test. The significant difference in demographic data (age, body mass, height, and BMI) between the three groups was tested with a one-way ANOVA. The overall effect of treatment, time, or the interaction between time and treatment was estimated using a 3 × 3 mixed-design multivariate analysis of variance (MANOVA). Wilks’ lambda was used to calculate the F value, and additional univariate ANOVAs (two-way mixed models) were carried out when the MANOVA showed a significant time group interaction effect. The alpha level of significance was set at *p* value ≤ 0.05. SPSS Version 23 was used to perform statistical analysis (SPSS, Inc., Chicago, IL, USA).

## 3. Results

A total of 75 patients were assessed for eligibility, of whom, 66 patients were randomly assigned to 1 of the 3 groups of intervention, and we obtained follow-up data from 60 (90.9%) patients. The study consort diagram is shown in Figure 1. No side effects attributable to the intervention were recorded.

### Demographic and Clinical Characteristics of the Patients

Baseline demographics and ulcer surface area and volume were not significantly different across patients (Table 1). Repeated measures MANOVA demonstrated a significant main effect of both time (Wilks’ λ = 0.08, F (4, 45) = 125.06, *p* = 0.0001, η^2^ = 0.91) and treatment (Wilks’ λ = 0.59, F (4, 94) = 6.91, *p* = 0.0001, η^2^ = 0.22), along with a substantial time × treatment interaction (Wilks’ λ = 0.23, F (8, 90) = 12.18, *p* = 0.0001, η^2^ = 0.52).

A significant change in ulcer surface area was observed in follow-up univariate ANOVAs, F (4, 96) = 40.07, *p* < 0.0001, η^2^ = 0.62, and for ulcer volume, F = (4, 96) = 41.12, *p* < 0.0001, η^2^ = 0.63. This means that the differences between groups on a linear combination of outcomes vary between pre-and post-intervention.

The between-group analysis revealed a significant difference in all measures (*p* < 0.05). The Bonferroni pairwise comparisons indicated that the three groups were significantly different, with the PEMF + PRE group exhibiting the most significant changes, where the average differences between the three groups (PEMF + PRE, PEMF, and control) at [95% confidence interval] for ulcer surface area were (−1.94, −1.48, −0.41, respectively) and for ulcer volume, (−4.62, −3.45, −1.10, respectively).

At the 4-week follow-up, the within-group analysis revealed a considerable variation in ulcer surface area and ulcer volume relative to baseline in the 2 experimental groups. The control group, however, showed no significant change (*p* > 0.05).

At the 12-week follow-up, the within-group analysis revealed a significant variation in ulcer surface area and ulcer volume compared to the baseline (*p* < 0.05).

**Figure 2:**
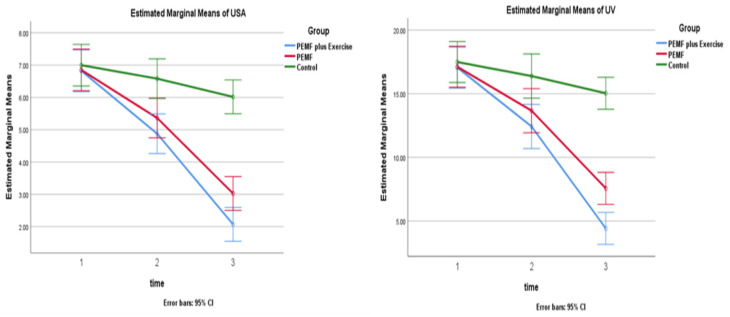
Baseline, 4-week, and 12-week differences in ulcer surface area (USA) and ulcer volume (UV) between groups.

The Bonferroni multiple comparison analysis revealed a significant difference between the three groups (PEMF + PRE, PEMF, and control) on the chosen parameters post-intervention, while the PEMF + PRE group underwent the most significant changes, with mean differences at [95% confidence interval] for ulcer surface area being (−4.75, −3.82, −0.98) and for ulcer volume (−12.63, −9.55, −2.45), respectively, Table 2 and Table 3, and Figure 2.

## 4. Discussion

According to the study’s findings, the control group did not experience any significant changes over the four-week follow-up period, while the two experimental groups showed a substantial improvement in ulcer surface area and volume. There were significant differences across the 3 groups at the 12-week follow-up, with the PEMF + PRE group experiencing the most significant improvement.

These results were in line with the findings of a study by El-Din et al. [29], which showed a reduced ulcer area of 61.2% for venous ulcers and 54.3% for vasculitic ulcers after 3 months of active treatment, pulsed electromagnetic field stimulation. Another study by Ieran et al. [31], observed significant wound healing in patients undergoing 75 Hz pulsed electromagnetic therapy after a 3-month treatment period: healing took an average of 71 days to complete. In a different study by Stiller et al. [32], patients with leg ulcers were treated with pulsed electromagnetic fields, and the active group showed a substantial increase in leg ulcer healing with a 47 % decrease in ulcer area, compared to the placebo group. Furthermore, in q study by Kenkre et al. [33], who used electromagnetic field therapy to treat long-standing venous leg ulcers resistant to conventional treatments, they found that 68% of the patients showed improvements in the size of their ulcers (4; 21% of which completely healed) (*p* < 0.05) throughout the trial. At day 50, it was discovered that patients receiving 800 Hz electromagnetic therapy recovered significantly faster than those receiving a placebo therapy (*p* < 0.05). Masoudi et al. [34] described two case reports of patients with refractory skin ulcers in two older and frail patients with several chronic skin ulcerations. Despite receiving the proper care, the ulcers made little progress, and the likelihood of an amputation being required was significant. However, when the patient resumed daily pulsed electromagnetic field therapy and received routine dressing, major improvements were shown, and within a few weeks of therapy, all ulcers had healed. Moreover, Caedo-Dorantes et al. [35] found that after exposure to low-frequency electromagnetic fields, the responders in their study had a healing velocity of 0.3–3 percent of their leg ulcers.

Several experimental studies have reported improved wound healing from various causes, including one that compared the histological and morphological differences between electric stimulation and magnetic field treatments in burn-injured rats. The pulsed electromagnetic field therapy group showed more healing signs (burn area, epithelialization, edema, and hyperemia) than the electric stimulation group [36]. Another experimental study by Athanasiou et al. [37], examining the short-term effects of PEMF on full-thickness skin injury, discovered a statistically significant increase in the healing rate in the experimental group during the first nine days, with histological inspection demonstrating a remarkable improvement in healing progression at all time points, compared to the control group. In the PEMF group, there was an improvement in epithelialization, collagenization, and angiogenesis under the microscopical assessment of injury healing. Another study examined the biomechanical changes that occurred when standard cutaneous wounds were exposed to pulsed radiofrequency magnetic fields under specific dose limits. It was observed that doing so quickened early wound healing, as shown by significantly higher wound tensile strength at 21 days following wounding [38]. An in vitro study by Costantini et al. [39] demonstrated that exposure to a low-frequency sinusoidal electromagnetic field quickened oral healing. This is most likely accomplished by an early increase in the proliferation of human gingival fibroblasts and the expression of the inflammatory mediators transforming growth factor beta 1, inducible nitric oxide synthase, and IL-6.

The pulsed electromagnetic field has several physiological impacts, which have been well-documented. It has been proposed that PEMF may induce particular, quantifiable biological reactions, such as DNA synthesis, transcription, and protein biosynthesis by modifying or enhancing existing endogenous electrical fields [40]. These biological reactions seem to occur in a range of PEMF settings. According to studies, PEMFs shorten the time it takes for fibroblasts to double in size and encourage skin fibroblast differentiation on culture [29], PEMF also increases angiogenesis, collagenization, and tissue regeneration [37], while enhancing wound tensile strength [38]. Other findings illustrated that electromagnetic field therapies could cause vasodilation and enhance peripheral blood flow, which has been proven to reduce inflammation and speed up cell proliferation [41]. Furthermore, PEMF has an impact on the immune system because it boosts the production of antibodies and circulatory neutrophils and increases polymorph nuclear leukocytes’ phagocytic activity [42].

One important element in the pathophysiology of venous ulcers is the increased generation of oxygen free radicals and lipid peroxides by both entrapped white cells due to decreased perfusion caused by increased venous pressure [43], as well as by cutaneous iron overload of extravasated red blood cells, leading to tissue destruction and endothelial damage [44]. In a recent study, PEMF was shown to decrease lipid peroxidation, raise antioxidant creation to enhance the endogenous defense system against free radicals and protect cells from cellular lysis and O_2_ toxicity [45]. All previously proven effects could be the underlying mechanism for improved wound healing by PEMF.

According to published research, there is a connection between calf muscle malfunction and the severity of VLU [46,47]. Two studies reported that isotonic resisted exercise enhances ejected venous volume, ejection fraction, and overall hemodynamic state in limbs with venous ulcers by raising the calf muscle’s muscular endurance, efficacy, and power [48,49]. A better calf muscle pump will increase venous pressure, promote blood flow, and ultimately, improve ulcer healing [50].

Several types of physical activity, such as resistance exercise, walking, aerobic exercise, and ankle exercise, are suggested as adjunct therapies to compression therapy for managing VLU [5,51,52]. According to a recent analysis, resistance training is the physical activity/exercise intervention that has been studied the most and is clinically effective for reducing ulcer size and increasing calf muscle pump function in people with VLU [53]. The results of the study are supported by findings from earlier studies; indeed, a recent study demonstrated that the use of a supervised exercise program (a combination of resistance, aerobic, and flexibility training) in conjunction with compression therapy was feasible and acceptable in the treatment of venous ulcers [54]. After 12 months, the exercise group’s healing rate was higher (83 percent vs. 60 percent), and the ulcer healing time was shorter [54]. Resistive training may result in persistent effects, such as calf muscle hypertrophy, increased blood flow, and vascular re-capillarization [55], all of which can improve ulcer healing.

### Strengths and Limitations

The findings of this study informed fundamental knowledge and offered a number of advantages, including its originality and use of various ulcer healing measures, such as ulcer surface and ulcer volume, as objective evaluations of ulcer healing. An additional strength is the design of the study, which includes randomization, blinding the assessors to the group allocation, and direct investigator monitoring of the intervention, which all increased the validity of the study’s findings. However, we would like to acknowledge some of the limitations, including the lack of long-term follow-up evaluations, which prevented us from analyzing the intervention’s long-term effects. Additionally, we did not assess ulcer size and area using more accurate methods, such as digital planimetry or jeltrate volume measurement. Moreover, the relatively small sample size in our study limits the generalizability of our findings. Therefore, future studies should aim to replicate our findings in larger and more diverse populations to enhance the generalizability of the results.

## 5. Conclusions

Combined plantar flexion resistance training and PEMF could be a non-invasive adjuvant treatment for venous leg ulcers and is proven to have more noticeable benefits on the healing process.

## Figures and Tables

**Table 1:** Demographic and clinical characteristics of subjects.

Variables	PEMF + PRE Group			PEMF Group			Control Group			*p*-Value
	Min	Max	Mean (SD)	Min	Max	Mean (SD)	Min	Max	Mean (SD)	
Age (years)	46.02	58.10	52.06 (3.02)	45.56	58.44	52 (3.22)	45.47	58.79	52.13 (3.33)	0.84
Sex (male/female)	9/6			6/9			7/8			0.56
Weight (kg)	52.20	91.80	72 (9.90)	53.54	98.46	76 (11.23)	54.08	95.92	75 (10.46)	0.31
Ulcer surface area (cm^2^)	4.14	9.50	6.82 (1.34)	4.44	9.24	6.84 (1.20)	4.19	9.79	6.99 (1.40)	0.92
Ulcer volume (cm^3^)	10.33	23.77	17.05 (3.36)	11.07	23.15	17.11 (3.02)	10.49	24.49	17.49 (3.50)	0.97

Abbreviation: PEMF, pulsed electromagnetic field; PRE, plantar flexion resistance exercise (PRE); SD, standard deviation. Level of significance at *p* < 0.05.

**Table 2:** Comparison of outcomes across the three groups at three-point timelines.

Characteristics		PEMF + PRE Group Mean (SD)	PEMF Group Mean (SD)	Control Group Mean (SD)	*p*-Value **		
					PEMF + PRE Group Vs. PEMF Group	PEMF + PRE Vs. Control	PEMF Vs. Control
Ulcer surface area (cm^2^)	Baseline	6.82 (1.34)	6.84 (1.20)	6.99 (1.40)	1.00	1.00	1.00
	4 weeks	4.87 (1.23)	5.36 (1.44)	6.57 (1.06)	0.79	0.001	0.02
	12 weeks	2.07 (1.15)	3.02 (1.05)	6.01 (0.99)	0.037	0.001	0.001
Ulcer volume (cm^3^)	Baseline	17.05 (3.36)	17.11 (3.02)	17.49 (3.50)	1.00	1.00	1.00
	4 weeks	12.42 (4.05)	13.66 (3.84)	16.39 (2.60)	0.94	0.006	0.01
	12 weeks	4.41 (2.05)	7.56 (3.08)	15.04 (2.48)	0.003	0.001	0.001

Abbreviation: PEMF, pulsed electromagnetic field; PRE, plantar flexion resistance exercise (PRE); SD, standard deviation; vs: versus. Level of significance at *p* < 0.05. ** *p*-value adjusted for pairwise multiple comparison: Bonferroni.

**Table 3:** Within-group pairwise comparisons at baseline, 4 weeks, and 12 weeks.

Characteristics		Baseline Vs. 4 Weeks		Baseline Vs. 12 Weeks	
		MD (95% CI)	*p*-Value **	MD (95% CI)	*p*-Value **
Ulcer surface area (cm^2^)	PEMF + PRE Group	−1.94 (−2.46, −1.42)	0.0001	−4.75 (−5.37, −4.12)	0.0001
	PEMF Group	−1.48 (−2.00, −0.96)	0.0001	−3.82 (−4.44, −3.19)	0.0001
	Control Group	−0.41 (−0.93, −0.10)	0.157	−0.98 (−1.60, −0.35)	0.001
Ulcer volume (cm^3^)	PEMF + PRE Group	−4.62 (−6.00, −3.24)	0.0001	−12.63 (−14.22, −11.05)	0.0001
	PEMF Group	−3.45 (−4.83, −2.06)	0.0001	−9.55 (−11.13, −7.96)	0.0001
	Control Group	−1.10 (−2.48, −0.28)	0.162	−2.45 (−4.03, −0.86)	0.001

Abbreviation: PEMF, pulsed electromagnetic field; PRE, plantar flexion resistance exercise (PRE); MD: mean difference; CI: confidence interval; vs: versus. Level of significance at *p* < 0.05. ** *p*-value adjusted for pairwise multiple comparison: Bonferroni.

## Data Availability

The corresponding author will provide the identified datasets used in the current study upon reasonable request.
